# Linoleic acid improves rosacea through repairing mitochondrial damage in keratinocytes

**DOI:** 10.1093/lifemedi/lnaf005

**Published:** 2025-02-23

**Authors:** Mei Wang, Wenqin Xiao, Tangxiele Liu, Yan Zhu, Mengting Chen, Zixin Tan, San Xu, Zhixiang Zhao, Fangfen Liu, Hongfu Xie, Xiang He, Zhili Deng, Ji Li

**Affiliations:** Department of Dermatology, Xiangya Hospital, Central South University, Changsha 410008, China; Hunan Key Laboratory of Aging Biology, Xiangya Hospital, Central South University, Changsha 410008, China; National Clinical Research Center for Geriatric Disorders, Xiangya Hospital, Central South University, Changsha 410008, China; Department of Dermatology, Xiangya Hospital, Central South University, Changsha 410008, China; Hunan Key Laboratory of Aging Biology, Xiangya Hospital, Central South University, Changsha 410008, China; National Clinical Research Center for Geriatric Disorders, Xiangya Hospital, Central South University, Changsha 410008, China; Department of Dermatology, The Affiliated Children’s Hospital of Xiangya School of Medicine, Central South University (Hunan Children’s Hospital), Changsha 410008, China; Department of Dermatology, Xiangya Hospital, Central South University, Changsha 410008, China; Hunan Key Laboratory of Aging Biology, Xiangya Hospital, Central South University, Changsha 410008, China; National Clinical Research Center for Geriatric Disorders, Xiangya Hospital, Central South University, Changsha 410008, China; Department of Dermatology, Xiangya Hospital, Central South University, Changsha 410008, China; Hunan Key Laboratory of Aging Biology, Xiangya Hospital, Central South University, Changsha 410008, China; National Clinical Research Center for Geriatric Disorders, Xiangya Hospital, Central South University, Changsha 410008, China; Department of Dermatology, Xiangya Hospital, Central South University, Changsha 410008, China; Hunan Key Laboratory of Aging Biology, Xiangya Hospital, Central South University, Changsha 410008, China; National Clinical Research Center for Geriatric Disorders, Xiangya Hospital, Central South University, Changsha 410008, China; Department of Dermatology, Xiangya Hospital, Central South University, Changsha 410008, China; Hunan Key Laboratory of Aging Biology, Xiangya Hospital, Central South University, Changsha 410008, China; National Clinical Research Center for Geriatric Disorders, Xiangya Hospital, Central South University, Changsha 410008, China; Department of Dermatology, Xiangya Hospital, Central South University, Changsha 410008, China; Hunan Key Laboratory of Aging Biology, Xiangya Hospital, Central South University, Changsha 410008, China; National Clinical Research Center for Geriatric Disorders, Xiangya Hospital, Central South University, Changsha 410008, China; Department of Dermatology, Xiangya Hospital, Central South University, Changsha 410008, China; Hunan Key Laboratory of Aging Biology, Xiangya Hospital, Central South University, Changsha 410008, China; National Clinical Research Center for Geriatric Disorders, Xiangya Hospital, Central South University, Changsha 410008, China; Department of Dermatology, Xiangya Hospital, Central South University, Changsha 410008, China; Hunan Key Laboratory of Aging Biology, Xiangya Hospital, Central South University, Changsha 410008, China; Department of Dermatology, The First Hospital of Changsha, Changsha 410005, China; The Affiliated Changsha Hospital of Xiangya School of Medicine, Central South University, Changsha 410008, China; Department of Dermatology, Shuguang Hospital Affiliated with Shanghai University of Traditional Chinese Medicine, Shanghai 201203, China; Department of Dermatology, Xiangya Hospital, Central South University, Changsha 410008, China; Hunan Key Laboratory of Aging Biology, Xiangya Hospital, Central South University, Changsha 410008, China; National Clinical Research Center for Geriatric Disorders, Xiangya Hospital, Central South University, Changsha 410008, China; Department of Dermatology, Xiangya Hospital, Central South University, Changsha 410008, China; Hunan Key Laboratory of Aging Biology, Xiangya Hospital, Central South University, Changsha 410008, China; National Clinical Research Center for Geriatric Disorders, Xiangya Hospital, Central South University, Changsha 410008, China

**Keywords:** rosacea, linoleic acid, PPARγ, mitochondrial damage

## Abstract

Rosacea, as a progressive and chronic inflammatory skin disease, lacks safe and effective treatment options. Our previous study reported metabolic disturbance in rosacea patients, containing abnormal lipid metabolism. Building on this, we characterized significant alterations in fatty acid metabolism among rosacea patients, with a notable increase in linoleic acid (LNA) levels. We further demonstrated that LNA prevents rosacea-like dermatitis in LL37-induced rosacea-like mouse model. Our evidence indicated that LNA hyperactivates PPARγ signaling in the epidermis, a phenomenon observed in both rosacea patients and mouse model. Inhibiting PPARγ rescued the effect of LNA in LL37-induced mice. Additionally, our *in vivo* and *in vitro* evidence strongly supported the presence of mitochondrial damage in the keratinocytes of rosacea. LNA stimulated PPARγ to reduce the reactive oxygen species production, increasing the generation of ATP and recovering mitochondrial membrane potential. Finally, through a prospective cohort study utilizing UK Biobank data and linkage disequilibrium score regression (LDSC) regression analysis, we further confirmed LNA levels are negatively related to the risk of rosacea, highlighting LNA as a promising therapeutic strategy for rosacea treatment.

## Introduction

Rosacea is a chronic, progressive inflammatory skin disorder, characterized by flushing, erythema, telangiectasia, papules/pustules, and hyperplasia on the central face [1]. Approximately 5.46% people worldwide and 3.48% of Chinese population suffer from this disease, imposing significant psychological burden [2, 3]. However, the therapeutic effect of most interventions is not satisfactory because of the pathogenesis uncertainty.

Current investigations have revealed that the pathogenic process of rosacea involves genetic components, along with a dysregulation of cutaneous immune, vascular, and nervous systems [4]. Recently, the importance of metabolic regulation in rosacea is becoming evident. Our previous study revealed significant metabolic disorders in rosacea patients, including carbohydrates, fatty acids (FAs), and amino acids. Some of the altered metabolites were proved to promote the neurovascular activities [5]. In addition, multiple meta-analyses suggested rosacea was correlated with dyslipidemia [6]. A prospective cohort study also indicated “Mediterranean diet” pattern, composed of polyunsaturated FAs (PUFAs), may decrease the risk of rosacea [7]. However, how aberrant lipid metabolism, especially FAs, regulates rosacea is still unclear.

Linoleic acid (LNA), an essential FA derived from plant and nuts oils, is the main *n*-6 PUFA in Western diets. It serves as the precursor substance of other *n*-6 PUFAs, such as arachidonic acid and docosahexaenoic acid [8]. It is reported that LNA has profound potential in disease prevention. For example, it reduces the risk of diabetes and cardiovascular disease [8, 9], and could also enhance CD8^+^ T cell antitumor immunity [10]. Historical evidence from the 20th century indicated that evening primrose oil, which contains 70%–74% LNA, had moisturizing, anti-inflammatory, and antioxidant effects on the skin [11]. Subsequent research demonstrated that oral or topical administration of LNA alleviates atopic dermatitis and psoriasis vulgaris, as well as promoting skin wound healing [12, 13]. LNA is involved in biosynthesis of ω-O-acylceramides, which is essential for maintaining skin barrier [14–16]. Moreover, LNA metabolism can suppress the inflammation induced by CD4^+^ and CD8^+^ T cells while enhancing the anti-inflammatory functions of Tregs in psoriasis [17]. Until now, there lacks proof of the role LNA plays in rosacea development and whether supplementing LNA could be served as a possible treatment option.

Here, we sought to elucidate the role of LNA in rosacea pathogenesis. Metabolomics revealed markedly enriched lipid metabolism in rosacea patients, especially LNA metabolic pathway. Our subsequent experiments demonstrated that elevated LNA prevented rosacea-like dermatitis in mice skin by enhancing the activity of PPARγ in keratinocytes. Moreover, after integrating data from human, mice, and cells, we reported severe mitochondrial damage and dysfunction in rosacea. LNA–PPARγ pathway improved rosacea by targeting mitochondrial damage. Finally, prospective cohort study and genetic analysis from UK Biobank further validated the role of LNA in reducing the risk of rosacea in the population. These findings revealed the protective effect LNA had on rosacea and shed some new light on its clinical intervention.

## Results

### LNA elevated in rosacea patients prevents the development of rosacea

**Figure 1.**
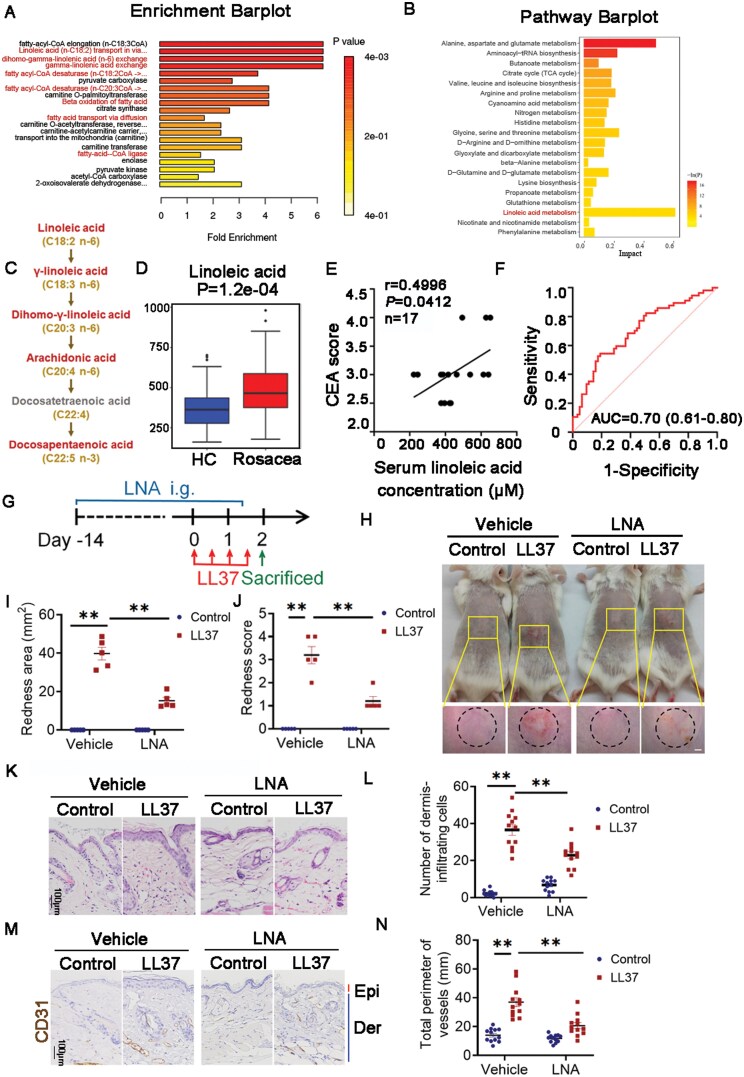
**LNA increased in serum of rosacea patients inhibited the development of disease.** (A) Pathway enrichment analysis of different metabolites between the healthy control and rosacea groups before and after treatment using pathway-associated metabolite sets. (B) Differential metabolite pathway analysis using the HSA set between healthy controls and rosacea groups before and after treatment. (C) Metabolites involved in LNA metabolism pathway. (D) Boxplot of LNA level between healthy control and rosacea patients. HC, healthy control (blue) (*n* = 63); Rosacea, rosacea patients (red) (*n* = 57). (E) Correlation between serum LNA levels and CEA scores in rosacea patients with mild to severe erythema. (F) Receiver operating characteristic [18] curve based on the serum LNA level in rosacea patients. AUC, area under the curve. (G) Schematic of LNA gavage administration for 16 consecutive days followed by intradermal LL37 injection in mice. Mice were sacrificed on Day 2 for subsequent experiments. (H) Back skin of mice treated with LNA or vehicle, followed by LL37 or PBS injections (*n* = 5 per group). Images were taken 48 h after the first LL37 injection. Scale bar: 2 mm. (I) Evaluation of rosacea-like phenotype severity based on redness area (*n* = 5). (J) Severity of rosacea-like phenotype based on redness score (*n* = 5). (K) Hematoxylin and eosin (HE) staining of skin sections from LNA or vehicle-treated mice injected with LL37 or PBS (*n* = 5). Scale bar: 100 μm. (L) Quantification of dermal infiltrating cells (*n* = 12) from high-power fields. (M) IHC of CD31 staining in lesional skin sections from mice (*n* = 12). Scale bar: 100 μm. (N) Calculation of blood vessel circumference for each group (*n* = 12). Data are representative of at least three independent experiments. Values represent mean ± SEM. *P < 0.05, **P < 0.01, ***P < 0.001. Spearman’s correlation test was used for (E), and one-way ANOVA with Bonferroni’s post hoc test was used for (I, J, L, and N).

To figure out FAs’ alteration in the peripheral blood of rosacea patients, we performed pathway enrichment analysis of serum metabolites between 57 rosacea patients before and after treatment, and 63 healthy controls as previously described [5]. The analysis of predicted metabolite sets identified multiple FA metabolism pathways involving LNA among the top 10, including fatty acyl-CoA desaturase, LNA (*n*-C18:2) transport via diffusion, carnitine O-palmitoyltransferase, beta-oxidation of FAs, mitochondrial transport, and carnitine transferase (Fig. 1A). KEGG pathway analysis demonstrated that LNA metabolism scores the highest impact value, which highlights its importance in rosacea (Fig. 1B). Among LNA metabolism, multiple metabolites were elevated such as LNA, γ-LNA, dihomo-γ-LNA, arachldonate, and docosapentaenoic acid (Fig. 1C). LNA, as the precursor of this metabolic pathway, can only be absorbed from external source and is decomposed into downstream substances. Therefore, we confer that the activation of LNA metabolism pathway is derived from the increased level of LNA in rosacea patients (Fig. 1D). We conducted a correlation analysis to explore the clinical implications of LNA. A positive correlation was found between LNA levels and the Clinician’s Erythema Assessment (CEA) scores in patients with moderate to severe erythema (*r* = 0.4996, *P* = 0.0412, Fig. 1E). The receiver operating characteristic [18] curve presented that LNA identifies rosacea with an area under curve (AUC) values of 0.70 (Fig. 1F). Collectively, these results indicated that LNA metabolism, particularly LNA, plays a critical role in the pathogenesis of rosacea.

To investigate the function of LNA in the development rosacea, 6-week-old BALB/c female mice were orally administrated with LNA for 16 continuous days and then were intracutaneous injected with cathelicidin LL37 on the last 2 days (Fig. 1G). Twelve hours after LL37 injection, vehicle group exhibited obvious rosacea-like dermatitis while LNA lightened redness score and area markedly (Fig. 1H–J). Histological analysis and immunohistochemical staining of CD31 indicated that LNA treatment markedly alleviated inflammatory cell infiltration and vasodilation in the dermis compared to the vehicle group (Fig. 1K–N). Therefore, high level of LNA relieves the pathological changes of rosacea, which indicates a promising strategy of disease.

### LNA blocks rosacea development by activating PPARγ in keratinocytes

**Figure 2.**
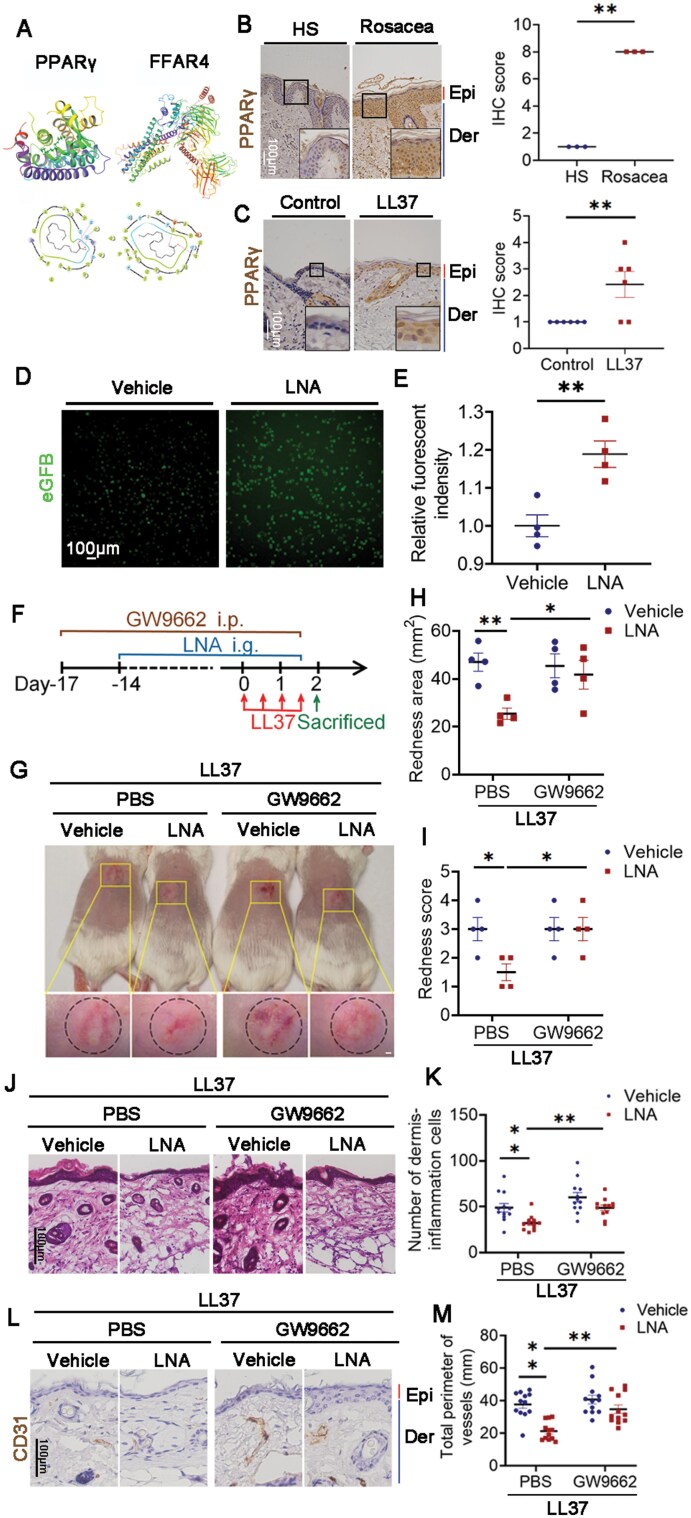
**LNA alleviated the progress of rosacea through PPARγ.** (A) Molecular docking analysis revealing the binding of LNA to its targets. (B) IHC analysis of PPARγ expression in skin sections from healthy and rosacea patients (*n* = 3). Higher magnification images of boxed areas are shown. Epi, epidermis; Der, dermis. Scale bar: 100 μm. (C) IHC analysis of PPARγ expression in skin sections from mice injected with LL37 or PBS (*n* = 6). Higher magnification images of boxed areas are shown. Epi, epidermis; Der, dermis. Scale bar: 100 μm. (D) Representative images showing PPARγ activity in HaCaT keratinocytes infected with PPRE–H2B–eGFP, treated with 25 μM LNA or vehicle for 2 h. Scale bar: 100 μm. (E) Quantification of fluorescence intensity in HaCaT keratinocytes after designated treatments (*n* = 4). (F) Schematic diagram of intraperitoneal GW9662 injection for 19 days and LNA gavage administration for 14 days in mice. Mice were injected with LL37 and sacrificed on Day 2 for further experiments. (G) Back skin of mice treated with LNA or vehicle, followed by intraperitoneal GW9662 or PBS injections (*n* = 4 per group). Images were captured 48 h after the first LL37 injection. Scale bar: 2 mm. (H) Evaluation of rosacea-like phenotype severity based on redness area (*n* = 4). (I) Evaluation of rosacea-like phenotype based on redness score (*n* = 4). (J) HE staining of skin sections from LNA or vehicle-treated mice with GW9662 or PBS (*n* = 12). Scale bar: 100 μm. (K) Quantification of dermal infiltrating cells (*n* = 12) for each high-power field. (L) IHC analysis of CD31 in lesional skin sections from mice (*n* = 12). Scale bar: 100 μm. (M) Calculation of blood vessel circumference for each group (*n* = 12). Data represents the mean ± SEM from at least three independent experiments. *P < 0.05, **P < 0.01, ***P < 0.001. Two-tailed unpaired Student’s *t*-test for (E), and one-way ANOVA with Bonferroni’s post hoc test for (H, I, K, and M).

It is generally believed that LNA produces a marked effect by activating FA receptors, such as PPARγ and FFAR4. Therefore, we predicted the interaction of LNA and PPARγ/FFAR4 using molecular docking to figure out the downstream pathway. Figure 2A presented that LNA binds to these two receptors by hydrogen bond, but Table 1 indicated docking score, glide gscore, and glide emodel of PPARγ are better than FFAR4’s. In order to determine the effector cells LNA activating PPARγ, immunohistochemistry (IHC) results indicated that PPARγ was mainly expressed in epidermis of rosacea patients and LL37-induced rosacea-like mice, while it showed little expression in healthy individuals or control mice (Fig. 2B and 2C). Fluorescence results of HaCaT keratinocytes indicated LNA strongly elevated the activity of PPARγ (Fig. 2D and 2E). For the sake of further proving the effect of PPARγ in the process of rosacea, GW9662, an inhibitor of PPARγ, was intraperitoneally injected to BALB/c mice. Mice were given LNA from the fourth day and injected with LL37 on the last 2 days as indicated (Fig. 2F). GW9662 reduced the activity raised by LNA (Fig. S1A). Result confirmed that GW9662 rescued the phenotype LNA exerted on LL37-induced mice, which means inhibiting PPARγ aggravates rosacea-like dermatitis alleviated by LNA (Fig. 2G–I). Meanwhile, GW9662 exacerbated dermis-inflammatory and vasodilation reduced by LNA in LL37 group (Fig. 2J–M). Taken together, PPARγ is hyperactivated by LNA in keratinocytes to relive rosacea.

**Table 1.** Result of molecular docking between LNA and PPARγ/FFAR4

Receptor	PDB ID	Docking score	Glide gscore	Glide emodel
PPARγ	6MS7	−6.234	−6.238	−62.562
FFAR4	8G59	−5.222	−5.226	−49.031

### Mitochondrial damage in keratinocytes accelerates rosacea development

**Figure 3.**
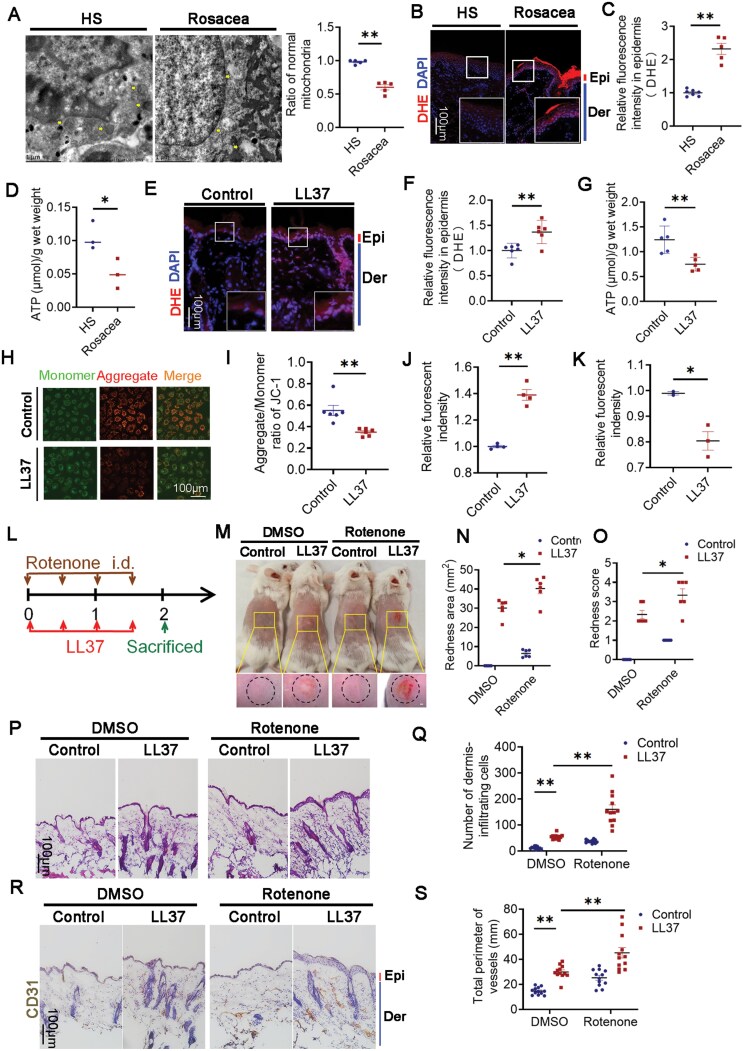
**Mitochondrial damage accelerated rosacea development.** (A) Transmission electron microscopy of keratinocytes in skin sections from healthy and rosacea patients (*n* = 5). Yellow arrows indicate mitochondria. Scale bar: 1 μm. (B) DHE staining of skin lesions from healthy controls and rosacea patients. Scale bar: 100 μm. (C) Quantification of relative fluorescence intensity for DHE in the epidermis (*n* = 5). (D) Quantification of ATP levels in lesional skin from healthy and rosacea patients (*n* = 3). (E) DHE staining of skin lesions from mice treated with LL37 or control vehicle. Scale bar: 100 μm. (F) Quantification of relative fluorescence intensity for DHE in epidermis (*n* = 6). (G) Quantification of ATP levels in lesional skin from mice treated with LL37 or control vehicle (*n* = 5). (H) JC-1 staining of HaCaT keratinocytes after 12-h LL37 stimulation. Scale bar: 100 μm. (I) Quantification of JC-1 fluorescence intensity in HaCaT keratinocytes after LL37 stimulation for 12 h (*n* = 6). (J) Quantification of ROS in HaCaT keratinocytes after LL37 stimulation for 12 h (*n* = 4). (K) Quantification of ATP in HaCaT keratinocytes after 24-h LL37 stimulation (*n* = 3). (L) Schematic of intradermal rotenone and LL37 injections for 2 days in mice. Mice were sacrificed on Day 2 for subsequent experiments. (M) Back skin of LL37 or control mice injected with rotenone or DMSO (*n* = 6). Images taken 48 h after the first LL37 injection. Scale bar: 2 mm. (N) Severity of rosacea-like phenotype evaluated based on redness area (*n* = 6). (O) Severity of rosacea-like phenotype evaluated based on redness score (*n* = 6). (P) HE staining of lesional skin sections from LL37 or control mice injected with rotenone or DMSO (*n* = 12). Scale bar: 100 μm. (Q) Quantification of dermal infiltrating cells (*n* = 12) from high-power fields. (R) IHC analysis of CD31 in lesional skin sections from mice (*n* = 12). Scale bar: 100 μm. (S) Calculation of blood vessel circumference for each group (*n* = 12). Data represent mean ± SEM from at least three independent experiments. *P < 0.05, **P < 0.01, ***P < 0.001. Two-tailed unpaired Student’s *t*-test for (C, D, F, G, and I–K) and one-way ANOVA with Bonferroni’s post hoc test for (N, O, Q, and S).

Next, we aimed to investigate specific mechanism LNA acts on rosacea. Previous studies suggested keratinocytes of rosacea presented oxidative stress and elevated level of reactive oxygen species (ROS) [19], which leads to irreversible cellular damage [20]. ROS are mainly produced in mitochondria while LNA was reported to reinforce mitochondrial bioenergetics and metabolic fitness of CD8^+^ T cell [10]. Accordingly, we supposed that LNA prevents rosacea development by improving mitochondrial function. To prove this, we observed the number and morphological changes of mitochondria in the epidermal of lesional skins from rosacea patients and controls by electron microscopy. The result indicated serious vacuolization and swelling of mitochondria accompanied by mitochondrial cristae and membrane disappearance (Fig. 3A). Mitochondria are the main place that generates ATP by oxidative phosphorylation, and are the major source of oxidative stress. Therefore, we evaluated ATP and ROS production in skin lesions of rosacea patients and controls. The results revealed that mitochondrial dysfunction brings about oxidative damage and impairs the production of ATP in epidermis (Fig. 3B–D). Subsequently, we identified similar phenomenon in rosacea-like mice model, i.e. upregulated ROS and decreased ATP level in keratinocytes (Fig. 3E–G). *In vitro*, LL37 induced the loss of mitochondrial membrane potentials and ATP level in HaCaT keratinocytes, as well as the enhancement of ROS level (Fig. 3H–K).

To explore the impact mitochondrial damage has on rosacea development, we treated rotenone and LL37 with mice for the sake of inducing mitochondrial disorder by inhibiting mitochondrial respiratory chain (Fig. 3L). Our findings suggested that rotenone exacerbated mitochondrial dysfunction, and promoted LL37-induced rosacea-like dermatitis, including inflammation and vasodilation (Figs. 3M–S, S2A–C). In a word, mitochondrial damage presented in epidermis accelerated the progress of rosacea.

### LNA recovers mitochondrial damage by activating PPARγ in keratinocytes of rosacea

**Figure 4.**
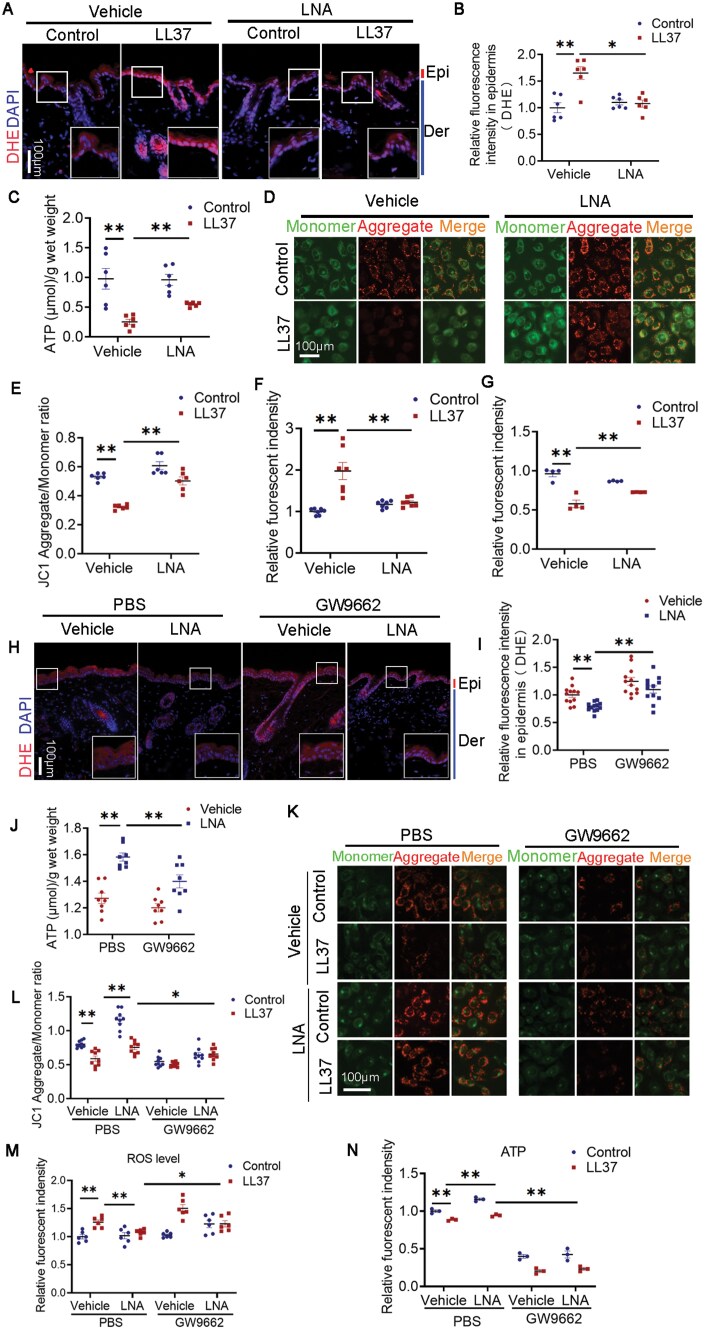
**LNA protected mitochondria from disorder by stimulating PPARγ.**(A) DHE staining of skin lesions from mice treated with LNA or vehicle and injected with LL37 or control vehicle. Scale bar: 100 μm. (B) Quantification of relative DHE fluorescence intensity in the epidermis (*n* = 6). (C) Measurement of ATP levels in lesional skin from LNA or vehicle-treated mice injected with LL37 or control vehicle (*n* = 6). (D) JC-1 staining in HaCaT keratinocytes after LNA treatment for 1 h followed by LL37 stimulation for 12 h. Scale bar: 100 μm. (E) Quantification of JC-1 fluorescence intensity in HaCaT keratinocytes after LNA treatment for 1 h and subsequent LL37 stimulation for 12 h (*n* = 6). Data are representative of at least three independent experiments. (F) Quantification of ROS production in HaCaT keratinocytes following LNA treatment for 1 h and LL37 stimulation for 12 h (*n* = 7). (G) Quantification of ATP levels in HaCaT keratinocytes following LNA treatment for 1 h and LL37 stimulation for 24 h (*n* = 4). (H) DHE staining of skin lesions from mice treated with LNA or vehicle and injected with GW9662 or PBS. Scale bar: 100 μm. (I) Quantification of DHE fluorescence intensity in epidermal tissue (*n* = 12). (J) Measurement of ATP levels in lesional skin from LNA or vehicle-treated mice injected with GW9662 or PBS (*n* = 8). (K) JC-1 staining of HaCaT keratinocytes after 12-h LL37 stimulation. Scale bar: 100 μm. (L) Quantification of JC-1 fluorescence intensity in HaCaT keratinocytes after LL37 stimulation for 12 h (*n* = 9). (M) Quantification of ROS levels in HaCaT keratinocytes after LL37 stimulation for 12 h (*n* = 6). (N) Quantification of ATP levels in HaCaT keratinocytes after LL37 stimulation for 24 h (*n* = 3). All results are representative of at least three independent experiments. Data represent the mean ± SEM. *P < 0.05, **P < 0.01, ***P < 0.001. One-way ANOVA with Bonferroni’s post hoc test (B, C, E–G, I, J, and L–N) was used.

To confirm whether LNA regulates mitochondrial homeostasis, we measured the ROS and ATP levels in the epidermis of LNA-treated LL37-induced mice. The data indicated that LNA downregulated ROS level increased by LL37 and upregulated ATP level reduced by LL37 (Fig. 4A–C). Likewise, in HaCaT keratinocytes, LNA administration restored mitochondrial membrane potentials or ATP production damaged by LL37 and improved oxidative stress induced by LL37 (Fig. 4D–G).

Moreover, we also verified whether LNA improves mitochondrial function through PPARγ. PPARγ inhibitor further aggravated oxidative stress and ATP dyssynthesis improved by LNA in epidermis (Fig. 4H–J). In line with *in vivo* experiments, blocking PPARγ reduced mitochondrial membrane potentials, upregulated ROS level, and lessen ATP level restored by LNA (Fig. 4K–N). Altogether, LNA stimulates PPARγ which recovers mitochondrial disorder in keratinocytes induced by LL37.

### LNA–PPARγ pathway improves rosacea via repairing mitochondrial damage

**Figure 5.**
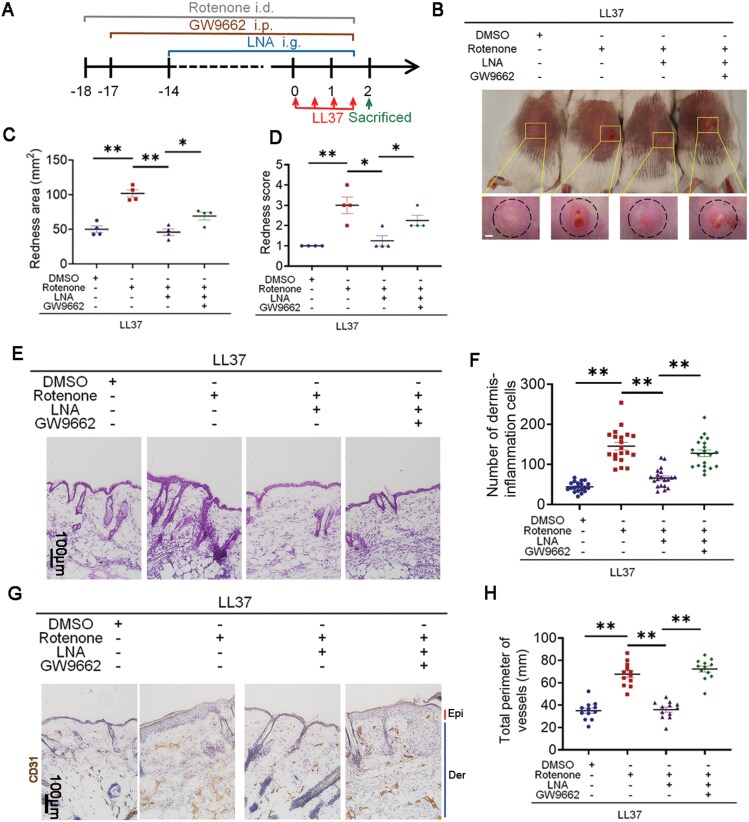
**LNA–PPARγ prohibited rosacea development via recovering mitochondrial function.** (A) Schematic representation of the treatment protocol: mice were injected with rotenone for 20 days, GW9662 for 19 days, and LNA via gavage for 16 days. Following 2 days of LL37 administration, mice were sacrificed for subsequent experiments. (B) Back skin images of LL37-treated mice treated with rotenone, LNA, GW9662, or a combination (*n* = 4). Images were captured 48 h after the first LL37 injection. Scale bar: 2 mm. (C) Assessment of rosacea-like phenotype severity based on redness area (*n* = 4). (D) Evaluation of rosacea-like phenotype based on redness score (*n* = 4). (E) HE staining of lesional skin sections from LL37-treated mice with rotenone, LNA, GW9662, or their combinations (*n* = 12). Scale bar: 100 μm. (F) Quantification of dermal infiltrating cells from each high-power field (*n* = 12). (G) IHC analysis of CD31 in lesional skin sections from mice (*n* = 12). Scale bar: 100 μm. (H) Calculation of blood vessel circumference in each group (*n* = 12). All results are representative of at least three independent experiments. Data represent the mean ± SEM. *P < 0.05, **P < 0.01, ***P < 0.001. One-way ANOVA with Bonferroni’s post hoc test (C, D, F, and H) was used.

To further prove LNA–PPARγ axis regulates rosacea development by affecting mitochondrial function, LL37-induced mice were treated with rotenone, LNA, and GW9662 (Fig. 5A). Mitochondrial function destroyed by rotenone was repaired through LNA and then damaged by GW9662 (Fig. S3A–C). Results indicated that rosacea-like dermatitis aggravated by rotenone was relieved by LNA, and then reversed by GW9662 (Fig. 5B–D). HE staining revealed infiltration of inflammatory cells was increased in rotenone group, decreased in LNA group with rotenone, and increased again after GW9662 treatment (Fig. 5E and 5F). Similarly, the diameter of dermal blood vessels in LNA group with rotenone was much smaller than rotenone group, but the diameter in GW9662 group was larger than LNA group (Fig. 5G and 5H). In conclusion, LNA–PPARγ signaling pathway restores mitochondrial function and thus heals rosacea.

### Prospective cohort study revealed the protective effect LNA had on rosacea patients

Afterwards, we validated the positive effect of LNA on rosacea patients in a cohort study. To explore the relationship between FAs and the risk of rosacea, we recruited 272,223 participants in UK Biobank database, with demographic information for the selected subjects provided in Table S1. During the follow-up period, 1318 participants developed rosacea. The associations between plasma FAs and the risk of rosacea in 1318 patients were displayed in Table 2. After fully adjusting the covariates, plasma PUFA was negatively correlated with risk of rosacea (Model 2, Q4 vs. Q1: 0.77, 95% CI: 0.66–0.91; hazard ratio (HR): 0.91, 95% CI: 0.86–0.97). Among them, *n*-6 PUFA and *n*-3 PUFA were associated with lower risk of rosacea (Model 2, Q4 vs. Q1: 0.81, 95% CI: 0.69–0.94; HR: 0.93, 95% CI: 0.87–0.98; Q4 vs. Q1: 0.79, 95% CI: 0.67–0.92; HR: 0.91, 95% CI: 0.86–0.97, respectively). Furthermore, the concentration of plasma LNA was significantly negatively related with risk of rosacea (Model 2, Q4 vs. Q1: 0.78, 95% CI: 0.67–0.92; HR: 0.94, 95% CI: 0.89–0.99).

**Table 2.** Associations between plasma FA and rosacea risk in UK Biobank (number of incident cases: 1318)

	Quartiles of plasma FA concentration				HR^a^ (95% CI)	*P*
	Q1	Q2	Q3	Q4		
SFA						
Cases/person-years						
Model 1	Ref.	1.00 (0.86, 1.17)	0.94 (0.80, 1.09)	0.93 (0.80, 1.09)	0.97 (0.91, 1.02)	0.222
Model 2	Ref.	0.99 (0.85, 1.16)	0.92 (0.79, 1.08)	0.91 (0.77, 1.06)	0.95 (0.90, 1.01)	0.114
MUFA						
Cases/person-years						
Model 1	Ref.	1.04 (0.89, 1.21)	1.00 (0.86, 1.17)	1.00 (0.85, 1.16)	0.99 (0.94, 1.05)	0.703
Model 2	Ref.	1.02 (0.88, 1.19)	0.97 (0.83, 1.14)	0.96 (0.81, 1.12)	0.97 (0.92, 1.03)	0.383
PUFA						
Cases/person-years						
Model 1	Ref.	0.92 (0.79, 1.07)	0.94 (0.81, 1.09)	0.78 (0.67, 0.91)	0.91 (0.86, 0.97)	0.002
Model 2	Ref.	0.92 (0.79, 1.07)	0.94 (0.80, 1.09)	0.77 (0.66, 0.91)	0.91 (0.86, 0.97)	0.002
*n*-6 PUFA						
Cases/person-years						
Model 1	Ref.	0.92 (0.79, 1.07)	0.98 (0.85, 1.14)	0.81 (0.69, 0.95)	0.93 (0.88, 0.98)	0.009
Model 2	Ref.	0.92 (0.79, 1.07)	0.98 (0.84, 1.14)	0.81 (0.69, 0.94)	0.93 (0.87, 0.98)	0.007
*n*-3 PUFA						
Cases/person-years						
Model 1	Ref.	0.92 (0.80, 1.07)	0.87 (0.75, 1.01)	0.79 (0.68, 0.93)	0.91 (0.86, 0.97)	0.002
Model 2	Ref.	0.91 (0.79, 1.06)	0.86 (0.74, 1.00)	0.79 (0.67, 0.92)	0.91 (0.86, 0.97)	0.002
LNA						
Cases/person-years						
Model 1	Ref.	0.89 (0.76, 1.03)	0.97 (0.84, 1.13)	0.78 (0.67, 0.92)	0.94 (0.89, 0.99)	0.029
Model 2	Ref.	0.89 (0.76, 1.03)	0.97 (0.84, 1.13)	0.78 (0.67, 0.92)	0.94 (0.89, 0.99)	0.030

^a^HR for per SD increment; Model 1, adjusted by age and sex; Model 2, additionally adjusted by Townsend deprivation index (quartiles), body mass index (BMI) (kg/m^2^: <18.5, 18.5–24.9, 25–29.9, 30–35, ≥35), smoking status (never, previous, current), alcohol intake frequency (never, <1 time/week, 1–4 times/week, daily), physical activity (MET (min/week): quartiles), education, income, vitamin and mineral supplementation.

Subsequently, we performed linkage disequilibrium score regression (LDSC) regression analysis in UK Biobank database to evaluate genetic correlation between FAs and rosacea. As presented in Table 3, *n*-6 PUFA (rg = −0.271, *P* = 0.045) and LNA (rg = −0.396, *P* = 0.021) showed a potential negative genetic association with rosacea while SFA, MUFA, and *n*-3 PUFA did not. Consequently, population data from UK Biobank supported that LNA plays a potential protective role in the process of rosacea.

**Table 3.** The linkage disequilibrium score regression results between FA and rosacea

Exposure	Genetic correlation (rg)	Standard error	*P-*value
*n*-3 PUFA	0.062	0.140	0.659
*n*-6 PUFA	−0.271	0.135	0.045
MUFA	−0.210	0.160	0.188
SFA	−0.200	0.149	0.179
PUFA	−0.273	0.156	0.081
LNA	−0.396	0.171	0.021

## Discussion

**Figure 6.**
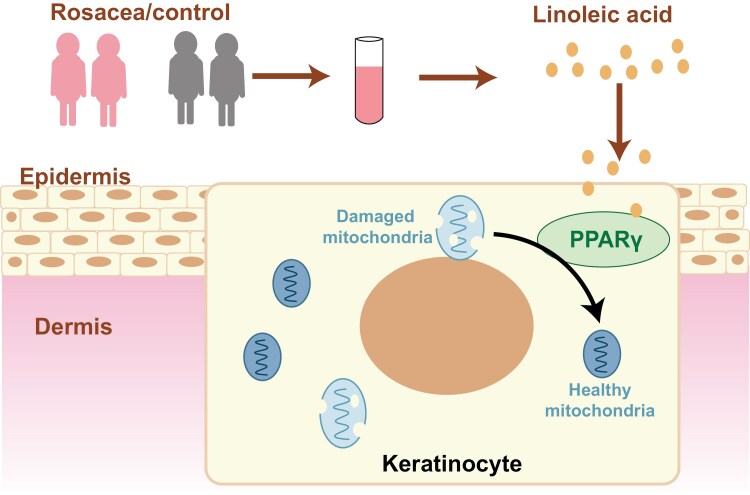
**Pattern diagram of how LNA activate PPARγ to prohibit rosacea development.** LNA levels were elevated in rosacea patients compared to healthy controls. By activating PPARγ, LNA facilitates the repairation of damaged mitochondria in keratinocytes at rosacea lesions, contributing to the inhibition of rosacea progression.

Serum metabolism reported enrichment of LNA metabolism pathway in rosacea patients and high level of LNA was related to the risk and severity of disease. Oral administration of LNA mitigated rosacea development, while inhibition of LNA receptor, PPARγ, promoted the development of rosacea-like lesions in mouse model. Mitochondrial damage was identified in the epidermis of lesions in rosacea patients, contributing to the skin inflammation of rosacea. LNA−PPARγ axis blocked rosacea pathogenesis by restoring mitochondrial function (Fig. 6). Prospective cohort study and LDSC regression analysis from UK Biobank further demonstrated plasma LNA may reduce the incidence of rosacea in large population.

FAs, a carboxylic acid with an aliphatic chain, are important energy source for the majority of human organs or tissues and main components of cell structures. Long-chain polyunsaturated FAs, containing omega-3 (*n*-3) and omega-6 (*n*-6) FAs, are defined as FAs have 12 or more carbons and one or more double bonds [21]. It is reported that PUFAs regulate systemic inflammation and participate in multiple inflammatory diseases, such as diabetes, obesity, arthritis, and ulcerative colitis [22]. Though *n*-3 FAs are generally considered anti-inflammatory and *n*-6 FAs pro-inflammatory, some studies have indicated that *n*-6 PUFAs improve insulin sensitivity and mitigate diabetic nephropathy [23, 24].

Sebum is a crucial component to maintain skin barrier integrity; therefore, FAs metabolic dysregulation may be the inducement of various skin diseases. In skin lesions of psoriasis patients, the free FAs are decreased, but monounsaturated FAs in blood are increased [25, 26]. FAs in skin lesions like arachidonic acid in atopic dermatitis patients are upregulated while serum DHA reduced largely [18, 27]. This evidence indicates both topical and systemic FA metabolism may be involved in skin diseases. Rosacea, an inflammatory skin condition, is associated with abnormal sebaceous FA composition, including reduced levels of long-chain saturated FAs in patients with papulopustular rosacea [28]. Moreover, a prospective cohort study among government employees reported Mediterranean-like diet pattern, characterized with high PUFAs intake, decreased the risk of rosacea [6]. It indicates abnormalities in blood lipids of rosacea but there still lacks corresponding research. Our study provides evidence for lipid metabolism disorders dominated by LNA metabolic pathway in rosacea.

LNA, the parent compound of *n*-6 PUFAs, is metabolized into γ-linolenic acid (GLA), dihomo-γ-linolenic acid (DGLA), arachidonic acid, eicosapentaenoic acid (EPA), and docosahexaenoic acid (DHA), all of which play key roles in various biological responses [29]. LNA reduces the risk of type 2 diabetes mellitus and coronary heart disease [8, 30]. Meanwhile, it constitutes 40% of FAs in the human skin and has a significant function in maintaining skin homeostasis. Cutaneous or systemic application of LNA helps repair impaired epidermal barrier function, initiate innate immune responses in acne, ameliorate atopic dermatitis, improve skin wound healing, and treat psoriasis [19, 29, 31–33]. However, hardly had study concentrated on the content and function of LNA in rosacea. Our result indicated rosacea patients acquire higher serum LNA which may be derived from diverse dietary habits of patients with more consuming of vegetable oils, nuts, and seeds. Our research also demonstrated LNA prevents rosacea development and is negatively associated with risk of disease, pointing out beneficial effect of LNA. Multiple countries and organizations suggested increasing LNA intake to protect against illness. For example, Food and Agriculture Organization of the United Nations recommends that *n*-6 FAs intake, mostly LNA, accounts for 5% of total energy intake [34]. Nevertheless, we also found LNA concentration was positively correlated with erythema in erythematotelangiectatic rosacea patients, which seems to be opposite to the protective role of LNA. We speculated that rosacea patients are accompanied by FA metabolic disorder. That is, enzymes oxidizing or decomposing LNA are hyperactivated in rosacea patients and transform high-level LNA to a large number of pro-inflammatory FAs, ultimately driving to severe erythema, but it needs further study to figure it out.

Apart from transforming to other substances to take part in energy metabolism, LNA regulates biological process by activating receptors like PPARγ and FFAR4 [23, 35]. Our data predicted PPARγ as LNA high affinity receptor and found that LNA promotes PPARγ activity. PPARγ, the most well-characterized member of the family of peroxisome proliferation-activated receptors, is mainly expressed in adipose tissue [35]. It strongly affected glucose metabolism, energy balance, and lipid biosynthesis. PPARγ, activated by endogenous ligands, works as transcription factor to increase expression of many target genes by forming heterodimerization with retinoid X receptors and binding to specific DNA response elements [36]. PPARγ is also widely expressed in skin, including keratinocytes, cutaneous immune cells, hair follicle, and sebaceous gland. It inhibits keratinocyte proliferation to regulate skin barrier permeability, stimulates the production of certain lipids, and represses the expression of pro-inflammatory genes [37]. Currently, various agonists were developed targeting PPARγ, such as the thiazolidinedione (TZD) family which may be helpful in psoriasis and atopic dermatitis, but it calls for more clinical evidence [37, 38].

There are studies reported that serum peroxide levels in rosacea patients were much higher than control subjects, while total antioxidative potential levels were markedly lower [39, 40]. Additionally, nanozymes treating oxidative stress and scavenging ROS improve treatment effect in rosacea-like mice [41]. These findings suggest systemic oxidative stress in rosacea. Our study provides direct evidence about disorders of mitochondrial morphology and function in keratinocytes of rosacea patients. It is widely acknowledged that mitochondrial dysfunction promotes inflammatory reaction by multiple approaches, including enabling the expression of pro-inflammatory genes or activating the NF-κB signaling pathway [42, 43]. Besides, mitochondria-derived ROS controls Ca(V)1.2 expression or activates Ca^2+^ sparks to dilate arteries [44, 45]. These studies provided underlying mechanism how mitochondrion takes part in the pathogenesis of rosacea. Research reported that supplementing the diet with LNA attenuates mitochondrial dysfunction in rats with hypertensive heart failure [46]. Moreover, LNA enhances mitochondrial content and activity of CD8^+^ T cell [10]. Our data demonstrated impaired mitochondrial function was reversed via LNA–PPARγ axis. Activated PPARγ interacts with PPARγ coactivator-1α (PGC1α) and regulates mitochondrial function, such as mitochondrial DNA replication, transcription and translation, TCA cycle, and so on [47, 48]. But the specific mechanisms that LNA–PPARγ affects mitochondrial function and the role of PGC1α in rosacea development remain unclear and need more explorations.

In conclusion, our study demonstrates that elevated serum LNA protects rosacea development via improving mitochondrial damage and suggests a promising intervention for rosacea.

## Research limitations

Admittedly, our data only reported the level of FAs in circulation, while did not measure the concentration of LNA in skin lesion of rosacea patients. We are currently working to collect more evidence to figure out the relationship between systemic metabolism state and local condition.

## Methods

### Patient sample collection and targeted metabolomics sequencing

Fifty-seven rosacea patients both before and after 8 weeks’ treatment, and 63 healthy controls, matched for BMI, gender, and age, were from the Department of Dermatology in Xiangya Hospital, Central South University. The inclusion and exclusion criteria, along with sample collection procedure, were performed as previously described [5].

Q300 Kit (Metabo-Profile, Shanghai, China) was applied in metabolomic analysis. All the identified metabolites were conducted KEGG pathway enrichment analysis, and compared using a hypergeometric test.

### Transmission electron microscopic examination

Skin biopsies from rosacea patients and healthy individuals were cut into 1 mm × 1 mm × 3 mm pieces, fixed in 2.5% glutaraldehyde solution with Millonig’s phosphate buffer (pH = 7.3), and were observed in the TEM laboratory at the Pathology Department of Xiangya Hospital, Changsha, Hunan. Samples were washed with Millonig’s phosphate buffer, and incubated for 1 h in 1% osmium tetroxide. Dehydration was done at room temperature by acetone with different concentrations. After resin embedding, the samples were polymerized overnight at 37°C, followed by solidification at 60°C for 12 h. Ultrathin sections (50–100 nm) were stained with lead nitrate and 3% uranyl acetate, and examined under a Hitachi HT-7700 electron microscope. Mitochondrial health was evaluated by counting normal mitochondria in randomly selected 5000× fields.

### Mice and treatments

BALB/c mice, with same sex and age, were obtained from Hunan SLAC Laboratory Animal Co., Ltd (Hunan, China). All mice were kept in specific pathogen-free (SPF) conditions with plenty of food and water. For LNA treatment, 6-week-old BALB/c mice were administered with 500 mg/kg per day of LNA (Sigma-Aldrich, USA) for 16 days by gastric perfusion. For PPARγ inhibitor (GW9662) treatment, mice were intraperitoneally injected with GW9662 (HY-16578, MCE) at a dose of 1 mg/kg per day for continuous 19 days. Control vehicle mice were treated with saline. For rotenone treatment, rotenone (10 µg, HY-B1756, MCE) or DMSO was topically applied to the back of mice along with LL37 injection every time. The LL37-induced rosacea-like dermatitis in mice was generated as before [49]. LL37 peptide in PBS (40 μL, 640 μM) or PBS was intradermally injected into the back skin of 8-week-old mice after hair shaving twice daily for 2 days. The mice were imaged and euthanized 48 h after the first application of LL37, and skin inflammation was assessed based on erythema severity. The redness score was rated on a scale of 1–5. The redness area was measured using a stereomicroscope (Leica S8AP0, Leica, Germany). Skin biopsy specimens were taken for IHC, histological analysis, and immunofluorescence.

### Histological analysis

Mouse samples were formalin-fixed and paraffin-embedded. Samples were sectioned into 5 μm slices and stained with hematoxylin and eosin (H&E). The features were evaluated by the infiltration of inflammatory cells in dermis, with three randomly selected microscopic areas (original magnification, 200×) per sample.

### Immunohistochemistry

Mouse skin samples were formalin-fixed and then paraffin-embedded. Five-micron sections were cut and incubated with primary antibody for CD31 (1:100, Cell Signaling, 77699s) and PPAR (1:100, Cell Signaling). Vessel perimeter, measured in three high-power fields per mouse (*n* = 6), was analyzed using ImageJ. IHC scores were assigned by staining intensity (0–3) and the percentage of positive cells (0–4). The averaged IHC score of epidermis was assessed for each human (*n* = 3) and each mouse (*n* = 6).

### Cell culture and treatment

HaCaT keratinocytes were obtained from NTCC (Biovector Science Lab, Beijing, China) and cultured in complete DMEM (Dulbecco’s Modified Eagle's medium) at 37°C. Upon 50% confluency, cells were starved overnight, and then incubated with LL37 (4 μM), LNA (25 μM), or GW9662 (10 μM) as indicated.

### Measurement of PPARγ activity

At 40%–50% confluency, HaCaT keratinocytes were starved for whole night and infected with PPRE–H2B–eGFP (Addggen) for 24 h. After treating cells with 25 μM LNA for 2 h, images were captured using a fluorescent microscope.

### Measurement of ROS generation

ROS levels were measured using DCFH-DA staining (Beyotime Biotech, Nantong, China). Cells (5 × 10^4^) were plated on 96-well plate. After exposure to either LL37, LNA, GW9662, or a combination for the indicated time, cells were stained with 10 μM DCFH-DA. Dichlorofluorescein (DCF) fluorescence was analyzed using spectrophotometer. ROS in skin sections was evaluated by dihydroethidium (DHE) staining. The frozen tissue sections were incubated with 5 μmol/L DHE (Beyotime, China) in PBS at 37°C for 30 min, followed by PBS washing. Fluorescence images were captured by a Zeiss microscope.

### Evaluation of mitochondrial membrane potential

Mitochondrial membrane potential was assessed through JC-1 staining (HY-15534, MCE). Cells were exposed to LL37, LNA, or GW9662 for indicated time. Fluorescence images were obtained by a Zeiss Axioplan 2 microscope.

### Evaluation of ATP level

Cell lysates or skin biopsy extracts were centrifuged, and ATP levels were quantified by a bioluminescent assay using an ATP assay kit (S0026, Beyotime).

### Molecular docking

The molecular structure of PPARγ (PDB ID: 6MS7) and FFAR4 (PDB ID: 8G59) was downloaded from PDB database. And the 3D structure of LNA ligand was downloaded from the PubChem database. Molecular docking analysis was performed by maestro software as previously described [50].

### LDSC analysis

We utilized genome-wide association studies (GWAS) summary data of FAs and rosacea-related phenotypes from the UK Biobank to assess their genetic correlation, using European linkage disequilibrium (LD) scores based on the 1000 Genomes as the linkage disequilibrium reference panel. A *P*-value of <0.008 (0.05/6, after Bonferroni correction) represents statistically significant; 0.008 < *P *< 0.05 was considered suggestive evidence of potential genetic correlation.

### Statistical analysis

Statistical analyses were performed with GraphPad Prism 6. After examining normal distribution and variance, differences were assessed by two-tailed unpaired Student's *t*-test for two groups and one-way ANOVA with Bonferroni’s post hoc test for multiple groups. Nonparametric tests (Whitney–Wilcoxon *U*-test, Kruskal–Wallis) were for non-normal data. Correlation analysis was performed by Pearson’s or Spearman’s *r*-test, as appropriate. Data are expressed as mean ± SEM.

### Research ethics

Human studies were approved by the ethical committee of the Xiangya Hospital, Central South University (IRB number 201703212). All participants signed written informed consent. Mice experiments were conducted based on the instructions of the ethical committee of the Xiangya Hospital, Central South University (IRB No. 201611610).

## Supplementary Material

lnaf005_suppl_Supplementary_Materials

## Data Availability

All data necessary to evaluate the conclusions of this study are provided in the manuscript or the Supplementary Materials. Any additional details supporting the findings of this study are available from the corresponding author upon reasonable request.
