# Supplementary material for: Linoleic acid improves rosacea through repairing mitochondrial damage in keratinocytes
Source: Life Med. 2025 Feb 23;4(2):lnaf005. doi: 10.1093/lifemedi/lnaf005 (PMC11971526; doi:10.1093/lifemedi/lnaf005)
Supplement: lnaf005_suppl_Supplementary_Materials [file lnaf005_suppl_supplementary_materials.docx]

**Linoleic acid improves rosacea through repairing mitochondrial damage in keratinocytes**

Mei Wang^1,2,3^, Wenqin Xiao^1,2,3^, Tangxiele Liu^4^, Yan Zhu^1,2,3^, Mengting Chen^1,2, 3^, Zixin Tan^1,2,3^, San Xu^1,2,3^, Zhixiang Zhao^1,2,3^, Fangfen Liu^1,2,3^, Hongfu Xie^1,2,5,6^, Xiang He^7^, Zhili Deng^1,2,3,*^, Ji Li^1,2,3,*^

^1^Department of Dermatology, Xiangya Hospital, Central South University, Changsha 410008, China

^2^Hunan Key Laboratory of Aging Biology, Xiangya Hospital, Central South University, Changsha 410008, China

^3^National Clinical Research Center for Geriatric Disorders, Xiangya Hospital, Central South University, Changsha 410008, China

^4^Department of Dermatology, The Affiliated Children's Hospital of Xiangya School of Medicine, Central South University (Hunan children’s hospital), Changsha, Hunan 410008, China

^5^The First Hospital of Changsha, Changsha 41005, China

^6^The Affiliated Changsha Hospital of Xiangya School of Medicine, Central South University, Changsha 410008, China

^7^Department of Dermatology, Shuguang Hospital Affiliated with Shanghai University of Traditional Chinese Medicine, Shangha 201203, China

^#^These authors contributed equally to this work.

Correspondence: liji_xy@csu.edu.cn (J.L.), dengzhili@csu.edu.cn (Z.D.)

Supplementary table 1. The demographic and clinical features of participants

| Characteristics | Total | Participants without rosacea | Participants newly diagnosed with rosacea | *P* | |
| --- | --- | --- | --- | --- | --- |
| Number | 272,223 | 270,905 | 1,318 |  | |
| Age (year, mean ± SD) | 56.6 ± 8.1 | 56.6 ± 8.1 | 56.1 ± 8.2 | 0.043 | |
| Sex |  |  |  | <0.001 | |
| Male | 125,465 (46%) | 124,943 (46%) | 522 (40%) |  | |
| Female | 146,758 (54%) | 145,962 (54%) | 796 (60%) |  | |
| Thomson deprivation index | −1.35 ± 3.08 | −1.35 ± 3.08 | −1.61 ± 2.92 | 0.002 | |
| Education |  |  |  | 0.001 | |
| College or university degree | 86,091 (39%) | 85,702 (39%) | 389 (37%) |  | |
| Professional qualification (e.g, nurses and teachers) | 14,143 (6.4%) | 14,048 (6.4%) | 95 (8.9%) |  | |
| A/AS level or equivalent education | 29,776 (13%) | 29,630 (13%) | 146 (14%) |  | |
| O/GCSE level or equivalent education | 58,131 (26%) | 57,831 (26%) | 300 (28%) |  | |
| Others | 33,141 (15%) | 33,008 (15%) | 133 (13%) |  | |
| Annual household income |  |  |  | 0.5 | |
| <£18,000 | 54,190 (23%) | 53,950 (23%) | 240 (22%) |  | |
| £18,000–£30,999 | 60,031 (26%) | 59,727 (26%) | 304 (27%) |  | |
| £31,000–£51,999 | 60,143 (26%) | 59,854 (26%) | 289 (26%) |  | |
| >£52,000 | 57,770 (25%) | 57,496 (25%) | 274 (25%) |  | |
| BMI，Kg/m^2^ | 27.46 ± 4.78 | 27.45 ± 4.78 | 27.62 ± 4.76 | 0.2 | |
| Smoking |  |  |  | 0.10 | |
| Never | 147,963 (55%) | 147,249 (55%) | 714 (54%) |  | |
| Past  Present | 94,192 (35%) | 93,711 (35%) | 481 (37%) |  | |
|  | 28,738 (11%) | 28,620 (11%) | 118 (9.0%) |  | |
| Drinking |  |  |  | | 0.3 |
| Never | 21,666 (8.0%) | 21,561 (8.0%) | 105 (8.0%) | |  |
| <once per week | 61,395 (23%) | 61,084 (23%) | 311 (24%) | |  |
| 1–4 times per week | 133,977 (49%) | 133,314 (49%) | 663 (50%) | |  |
| Everyday | 54,630 (20%) | 54,392 (20%) | 238 (18%) | |  |
| Physical activity (MET)，min/week | 2669.3 ± 2736.6 | 2669.6 ± 2737.5 | 2604.9 ± 2529.0 | | 0.4 |
| Vitamin supplement | 85,793 (32%) | 85,405 (32%) | 388 (30%) | | 0.11 |
| Minerals supplement | 33,552 (12%) | 33,409 (12%) | 143 (11%) | | 0.11 |

BMI：Body mass index


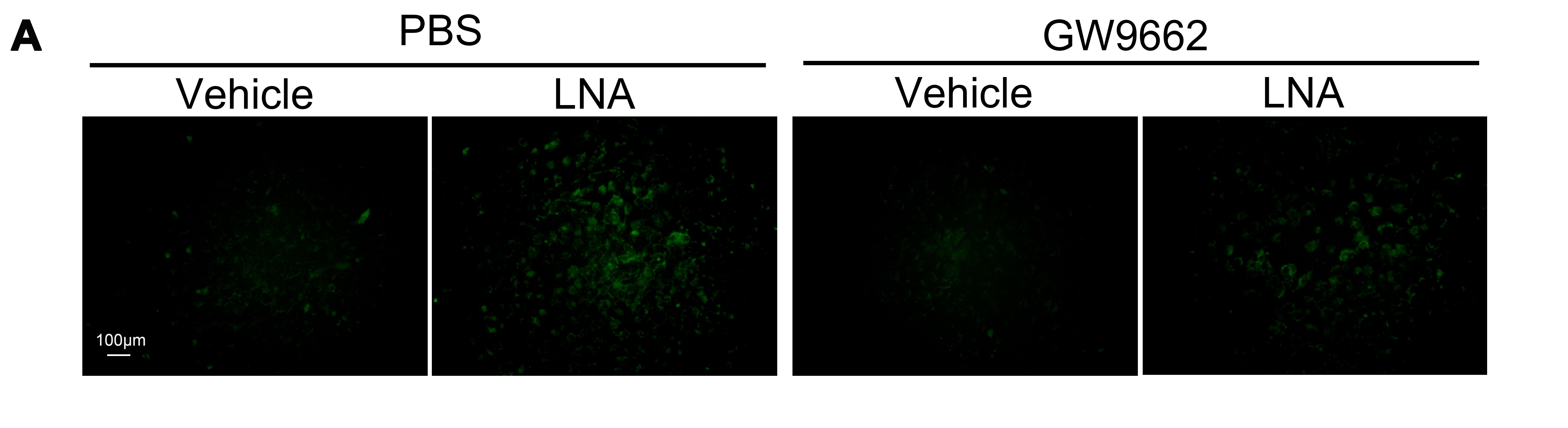


**Supplementary figure 1. PPARγ inhibitor decreased the activity of PPARγ.**

Representative images showing the activity of PPARγ analyzed by fluorescent microscope in live HaCaT keratinocytes infected with PPRE-H2B-eGFP for 24 h, and then treated with LNA and GW6992 for 2 h followed by PBS wash. Scale bar: 100 μm.


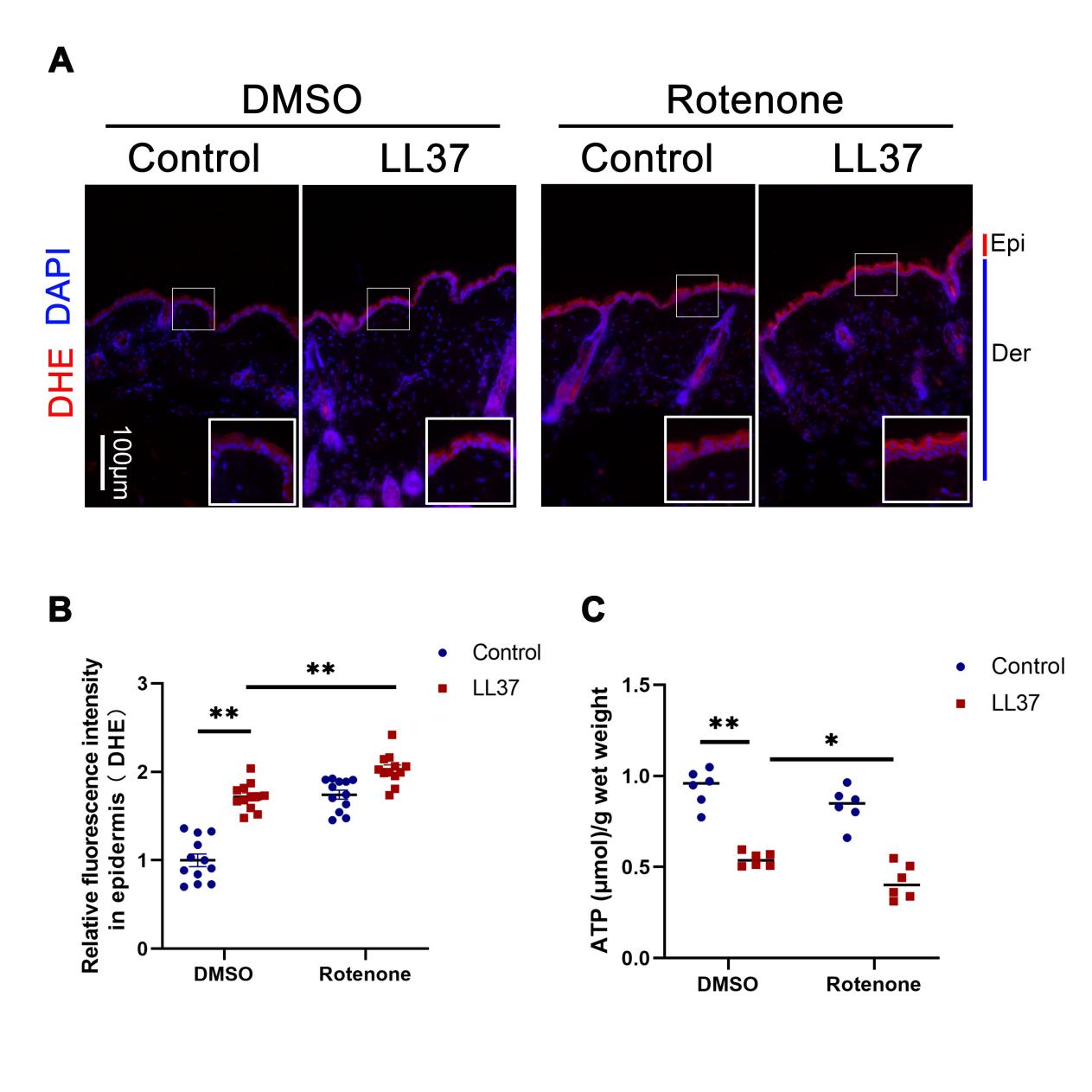


**Supplementary figure 2. Rotenone promoted mitochondrial damage.**

The DHE staining of skin lesion from LL37 or control mice injected with rotenone or DMSO. Scale bar: 100 μm. (**B**) Quantification of relative fluorescence intensity for DHE in epidermis (*n* = 12). (**C**) Quantification of ATP from lesional skin of LL37 or control mice injected with rotenone or DMSO. (*n* = 6). All results are representative of at least three independent experiments. Data represent the mean ± SEM. **P* < 0.05, ***P* < 0.01, ****P* < 0.001. One-way ANOVA with Bonferroni’s *post hoc* test (B and C) was used.

**
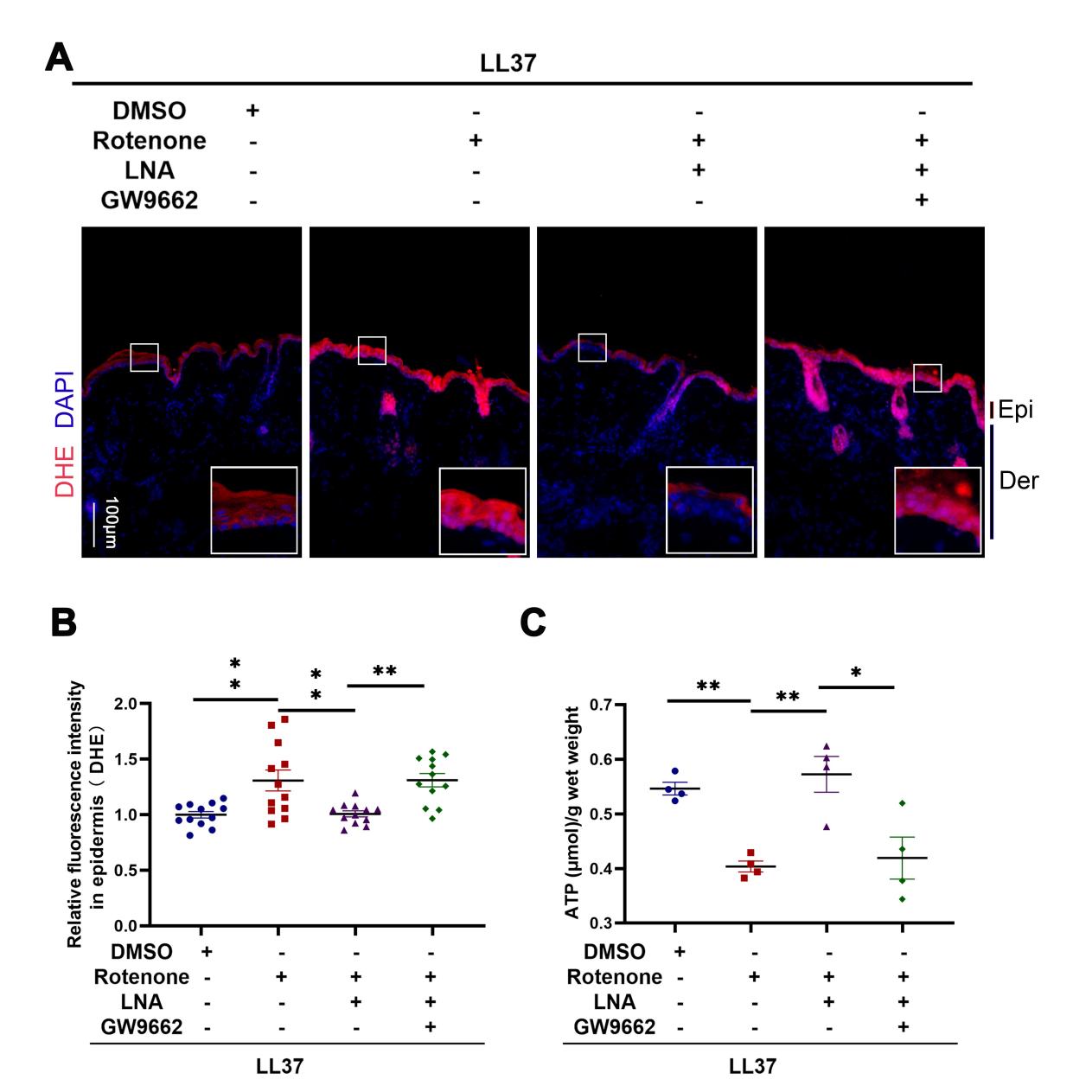
**

**Supplementary figure 3. LNA-PPARγ prohibited mitochondrial damage.**

1. The DHE staining of skin lesion from LL37 mice treated with rotenone, LNA, GW9662 or their combination. Scale bar: 100 μm. (**B**) Quantification of relative fluorescence intensity for DHE in epidermis (*n* = 12). (**C**) Quantification of ATP from lesional skin of LL37 or control mice injected with rotenone or DMSO. (*n* = 4). All results are representative of at least three independent experiments. Data represent the mean ± SEM. **P* < 0.05, ***P* < 0.01, ****P* < 0.001. One-way ANOVA with Bonferroni’s *post hoc* test (B and C) was used.
